# Transformable hybrid semiconducting polymer nanozyme for second near-infrared photothermal ferrotherapy

**DOI:** 10.1038/s41467-020-15730-x

**Published:** 2020-04-20

**Authors:** Yuyan Jiang, Xuhui Zhao, Jiaguo Huang, Jingchao Li, Paul Kumar Upputuri, He Sun, Xiao Han, Manojit Pramanik, Yansong Miao, Hongwei Duan, Kanyi Pu, Ruiping Zhang

**Affiliations:** 1 School of Chemical and Biomedical Engineering, Nanyang Technological University, 70 Nanyang Drive, Singapore, 637457 Singapore; 2 The Affiliated Bethune Hospital of Shanxi Medical University, Taiyuan, Shanxi 030032 People’s Republic of China; 3 School of Biological Science, Nanyang Technological University, Singapore, 637551 Singapore

**Keywords:** Biomedical materials, Nanoparticles, Nanotechnology in cancer

## Abstract

Despite its growing promise in cancer treatment, ferrotherapy has low therapeutic efficacy due to compromised Fenton catalytic efficiency in tumor milieu. We herein report a hybrid semiconducting nanozyme (HSN) with high photothermal conversion efficiency for photoacoustic (PA) imaging-guided second near-infrared photothermal ferrotherapy. HSN comprises an amphiphilic semiconducting polymer as photothermal converter, PA emitter and iron-chelating Fenton catalyst. Upon photoirradiation, HSN generates heat not only to induce cytotoxicity but also to enhance Fenton reaction. The increased ·OH generation promotes both ferroptosis and apoptosis, oxidizes HSN (42 nm) and transforms it into tiny segments (1.7 nm) with elevated intratumoral permeability. The non-invasive seamless synergism leads to amplified therapeutic effects including a deep ablation depth (9 mm), reduced expression of metastasis-related proteins and inhibition of metastasis from primary tumor to distant organs. Thereby, our study provides a generalized nanozyme strategy to compensate both ferrotherapy and phototherapeutics for complete tumor regression.

## Abstract (web summary)

Due to tumour microenvironment, Fenton reactions have low therapeutic efficiency. Here the authors report on the application of NIR-II hybrid semiconducting nanozymes for combined photothermal therapy and enhanced ferrotherapy with photoacoustic imaging and show application in vivo in tumour models.

## Introduction

Ferrotherapy that utilizes iron ions to catalytically disproportionate H_2_O_2_ to cytotoxic hydroxyl radical (·OH) holds promise for cancer treatment^1^. Because ferrotherapy mechanistically bypasses the drug resistance issue of classical chemodrugs, increasing endeavors are devoted to development of ferro-therapeutic agents^2,3^. However, due to unfavorable catalytic conditions in tumor microenvironment, current ferro-therapeutic agents suffer from poor therapeutic efficacy and thus are often combined with other therapeutic modalities^4^. For instance, iron-based nanomaterials have been delivered with chemotherapeutics (e.g. cisplatin)^5^, sonosensitizers (e.g. porphyrin)^6^, genetic materials (e.g. p53 plasmid)^7^, or immune checkpoint inhibitors (e.g. Nivolumab)^8^ for combinational chemotherapy, sonodynamic therapy, gene therapy, or immunotherapy, respectively. Despite their overall improved therapeutic outcomes, these combinational strategies lack synergy to overcome the intrinsic drawback of ferrotherapy to promote suboptimal catalytic efficiency in tumor milieu. In this regard, glucose oxidase has been employed in ferrotherapy to enhance Fenton reaction by elevating H_2_O_2_ supply^9^, whereas the non-specific H_2_O_2_ production in other tissues could cause side effects such as systemic inflammation.

Photothermal therapy (PTT) that utilizes chromophores to convert incident light into hyperthermia provides a non-invasive way to eradicate tumors^10^. For instance, PTT mediated by gold nanoshells has been utilized for high-precision ablation of prostate cancer in clinical trial without causing deleterious effects to organ function^11^. In addition, photoirradiation was reported to enhance delivery of doxorubicin to centimeter-scale depth in large solid tumor^12^. Because light in the second near-infrared (NIR) (NIR-II, 1000–1300 nm) window has higher maximum permissible energy to skin (1 W cm^−2^ for 1064 nm while 0.33 W cm^−2^ for 808 nm) and reduced tissue attenuation than that in the first NIR window (NIR-I, 650–950 nm)^13,14^, NIR-II absorbing PTT agents have been actively under development such as plasmonic gold blackbodies^15^, copper sulfide nanostructures^16^, platinum-derived nanoparticles^17^, niobium carbide^18^ etc. According to Arrhenius equation, exogenous heat can supply as a driving force to accelerate chemical reaction^19^. Specifically, kinetics studies indicate that Fenton reaction rate is enhanced by up to 4-fold upon increasing the temperature from 20 to 50 °C^20^. Thereby, PTT is promising to promote intratumoral Fenton reaction efficiency during ferrotherapy^21^. However, nanoagents that synergize NIR-II photothermal effect to augment ferro-therapeutic effect have been rarely reported^22^.

The major challenge for NIR-II photothermal ferrotherapy lies in the development of delivery systems that can act as both the iron chelator and NIR-II chromophore. Semiconducting polymer nanoparticles (SPNs) composed of highly *π*-conjugated backbones have emerged as a family of optical materials^23–25^. By virtue of the tunable photophysical property, chemical flexibility, and good biocompatibility, SPNs have been extensively exploited to convert light for therapeutic and biological interventions^26,27^, or developed into activatable probes for ultrasensitive molecular imaging^28,29^. In particular, band gaps of SPNs can be facilely modulated to afford excellent photothermal performance even in the NIR-II window^30^, allowing for non-invasive deep-tissue photoacoustic (PA) imaging of brain vasculature^31,32^, in vivo PA tracking of human mesenchymal stem cells^33^, phototherapeutics of deep tumor^34^, etc. Moreover, due to the abundant existence of sulfur and nitrogen atoms in the semiconducting backbone that have binding affinity towards metal ions^35,36^, certain structure units of SPs are known to be able to chelate metal ions, which have been utilized for metal ion sensing^36–38^. These previous studies clearly testify that rational design of SPNs is a promising approach towards NIR-II photothermal ferrotherapy.

**Fig. 1:**
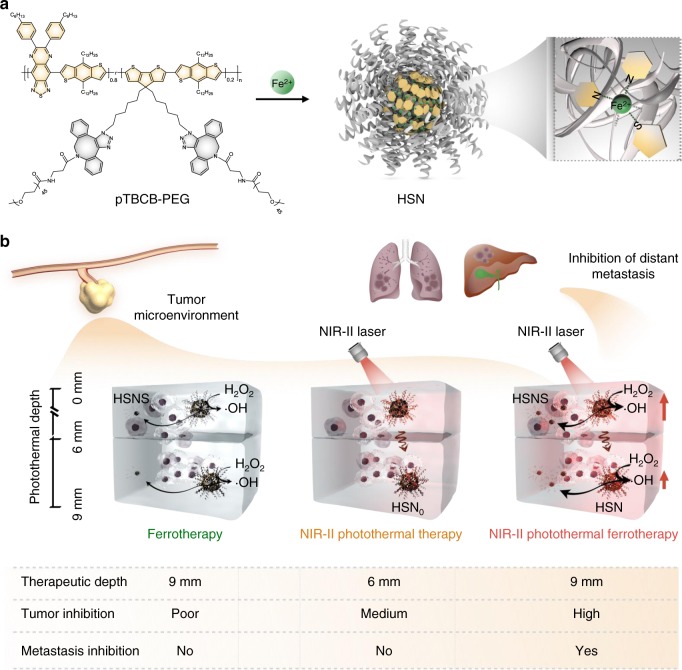
Schematic illustration of HSN for NIR-II photothermal ferrotherapy. **a** Chemical structure of pTBCB-PEG, preparation of HSN, and proposed mechanism of iron chelation. **b** Comparison of HSN-mediated NIR-II photothermal ferrotherapy with monotherapies (ferrotherapy and PTT).

Herein, we report the first hybrid semiconducting nanozyme (HSN) with photoirradiation enhanced catalytic activity for NIR-II PA imaging-guided synergistic photothermal ferrotherapy. An amphiphilic semiconducting polymer, PEGylated poly[(thiadiazoloquinoxaline-*alt*-benzodithiophene)-*ran*-(cyclopentadithiophene-*alt*-benzodithiophene)] (pTBCB-PEG), is synthesized to serve as both the NIR-II photothermal transducer and iron chelator. The chelation ability of pTBCB-PEG is originated from the backbone sulfur and nitrogen atoms that have high binding affinity towards ferrous ions (Fig. 1a). Upon NIR-II photoirradiation, pTBCB mediates photothermal transduction, which not only triggers PTT but also potentiates Fenton reaction to enhance both apoptosis and ferroptosis. Furthermore, the elevated ·OH production accelerates decomposition of HSN and thus transforms it to tiny fragments (~1.7 nm), favoring deep permeation into solid tumor for elevated antitumor effect in a photothermal depth-independent manner (Fig. 1b). As such, the seamless synergism of NIR-II photothermal ferrotherapy leads to tumor elimination at a remarkably deep-tissue depth in a non-invasive manner, contributing to complete cancer remission and metastasis inhibition.

## Results

### Synthesis and in vitro characterization

The NIR-II light-harvesting polymer precursor pTBCB(-Br) was synthesized via Stille polycondensation of 3 monomers: 4,9-dibromo-6,7-bis(4-hexylphenyl)-[1,2,5]thiadiazolo[3,4-g]quinoxaline (TDQ), (4,8-didodecylbenzo[1,2-b:4,5-b′]dithiophene-2,6-diyl)bis(trimethylstannane) (BDT), and 2,6-dibromo-4,4-bis(6-bromohexyl)-4H-cyclopenta[2,1-b:3,4-b′]dithiophene (CPDT-Br, Supplementary Figs. 1–3). Among these monomers, TDQ with strong electron-withdrawing ability was the acceptor to narrow the band gap; BDT was the donor to finetune the absorption spectrum; whereas CPDT-Br was a modifiable moiety for side chain post-functionalization. pTBCB-Br was converted to pTBCB-N_3_ through substitution of bromide with azide, and then grafted with methoxypolyethylene glycol dibenzocyclooctyne (DBCO-mPEG2000) via copper-free click chemistry to obtain pTBCB-PEG (Supplementary Figs. 2, 4). The ideal amphiphilicity of pTBCB-PEG allowed it to spontaneously self-assemble into nanoparticle termed as HSN_0_ in aqueous solution (Supplementary Fig. 5). Meanwhile, due to the presence of ion binding sites of pTBCB-PEG, the nanozyme HSN was facilely prepared by simple addition of ferrous ion into pTBCB-PEG solution during self-assembly.

**Fig. 2:**
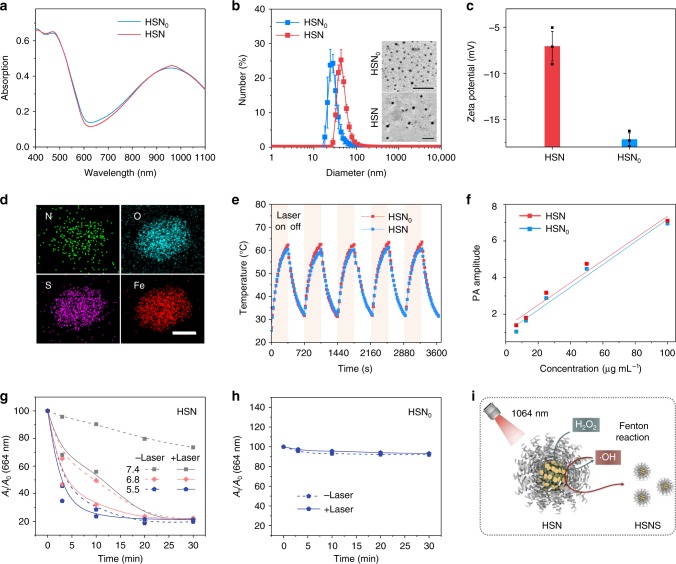
In vitro characterization of HSN. **a** Absorption spectra of HSN and HSN_0_ in 1 × PBS ([pTBCB] = 3 µg mL^−1^). **b** DLS profiles of HSN and HSN_0_ in 1 × PBS. *Inset*: TEM images of HSN_0_ and HSN. Scale bar: 200 nm. **c** Zeta potential profiles of HSN and HSN_0_. **d** STEM-EDX element mapping of HSN. Scale bar: 20 nm. **e** Photothermal heating-cooling cycles of HSN and HSN_0_ ([pTBCB] = 12 µg mL^−1^) under 1064 nm photoirradiation (1 W cm^−2^). **f** Linear fits of PA amplitudes of HSN (*R*^2^ = 0.96809) and HSN_0_ (*R*^2^ = 0.97314) as a function of concentration at 1064 nm, respectively. **g** ·OH generation of HSN under different pH conditions (pH = 7.4, 6.8, 5.5) with or without 1064 nm photoirradiation (1 W cm^−2^). ·OH generation was quantified by the decrease of absorbance of MB at 664 nm. [pTBCB] = 23 µg mL^−1^, [H_2_O_2_] = 0.5 mM, [MB] = 1 mM. **h** ·OH generation by HSN_0_ at pH 6.8 with or without 1064 nm photoirradiation (1 W cm^−2^). **i** Scheme of NIR-II photoirradiation enhanced self-degradation of HSN to HSNS in the presence of H_2_O_2_. Error bars indicated standard deviations of three independent measurements.

Both HSN and the iron-free control (HSN_0_) had maximum absorbance at 964 nm (Fig. 2a), showing that iron chelation had negligible effect on light-harvesting property. However, dynamic light scattering (DLS) indicated a larger hydrodynamic diameter for HSN (42 nm) relative to HSN_0_ (32 nm) (Fig. 2b). Transmission electron microscope (TEM) further confirmed the larger dimension of HSN and indicated the homogenous sphere-like morphology for both nanoparticles (Fig. 2b). There are negligible changes in diameter for both nanoparticles after storage for 2 months, implying their excellent colloidal stability (Supplementary Fig. 6). In addition, zeta potential measurement indicated the neutralization of surface charge from −17 mV for HSN_0_ to −7 mV for HSN (Fig. 2c). Scanning transmission electron microscopy-energy dispersive X-ray (STEM-EDX) element mapping illustrated that iron was distributed evenly across HSN (Fig. 2d, Supplementary Fig. 7). Quantitative analysis further indicated the respective atomic ratio of Fe (9.79%), N (11.49%), and S (7.14%).

Photothermal transduction capability of HSN was evaluated and compared with HSN_0_. Upon photoirradiation at 1064 nm, both nanoparticles induced significant aqueous temperature rise (Fig. 2e). After continuous irradiation for 360 s, the maximal solution temperature of HSN (62.3 °C) was slightly higher than that of HSN_0_ (60.5 °C). Furthermore, HSN had a higher photothermal conversion efficiency (PCE) (98.9%) than HSN_0_ (83.3%) (Supplementary Fig. 8), which to the best of our knowledge is the highest among all the NIR-II photothermal agents reported so forth^15^. The higher PCE for HSN was probably because of the enhanced heat transfer rate assigned to its larger size and increased intermolecular interactions due to iron chelation^39–42^. Correspondingly, HSN emitted higher PA amplitude than HSN_0_ at identical concentration (e.g. 1.3-fold at 6.5 µg mL^−1^) upon irradiation of pulsed 1064 nm laser (Fig. 2f). Furthermore, both HSN_0_ and HSN showed nearly unchanged photothermal performance during five heating-cooling cycles, indicating their excellent photo-stabilities.

The catalytic activities of HSN to generate ·OH were studied in pH conditions mimicking both physiological condition (pH 7.4) and tumor milieu (pH 6.8 at tumor microenvironment and pH 5.5 at lysosomes), respectively. Based on the well-known ·OH-induced bleaching of methylene blue (MB), the catalytic efficiency of HSN was quantified by measurement of the absorption decrease of MB at 664 nm. In the presence of H_2_O_2_ (0.5 mM), the decomposition of MB at pH 5.5 mediated by HSN was the fastest, followed by pH 6.8 and 7.4 (Fig. 2g). Although HSN_0_ hardly induced MB degradation under the same condition (Fig. 2h). After 3 min of photoirradiation, MB bleaching by HSN was, respectively, enhanced from 5 to 32% at pH 7.4, 35 to 53% at pH 6.8, and 55 to 65% at pH 5.5, suggesting that HSN-mediated photothermal heating significantly accelerated Fenton reaction rate. Along with ·OH generation, decreased absorbance of pTBCB-PEG at 960 nm was observed (Supplementary Fig. 9). DLS measurement indicated a decreased diameter of HSN from 42 to 1.7 nm after photoirradiation (Supplementary Fig. 10). Consistently, TEM illustrated the decomposition of HSN to form tiny segments (named as HSNS) (Supplementary Fig. 10, Fig. 2i). Further, gel permeation chromatography (GPC) proved much smaller molecular weight of HSNS relative to original polymer (Supplementary Fig. 9).

### In vitro NIR-II photothermal ferrotherapy and therapeutic mechanism

**Fig. 3:**
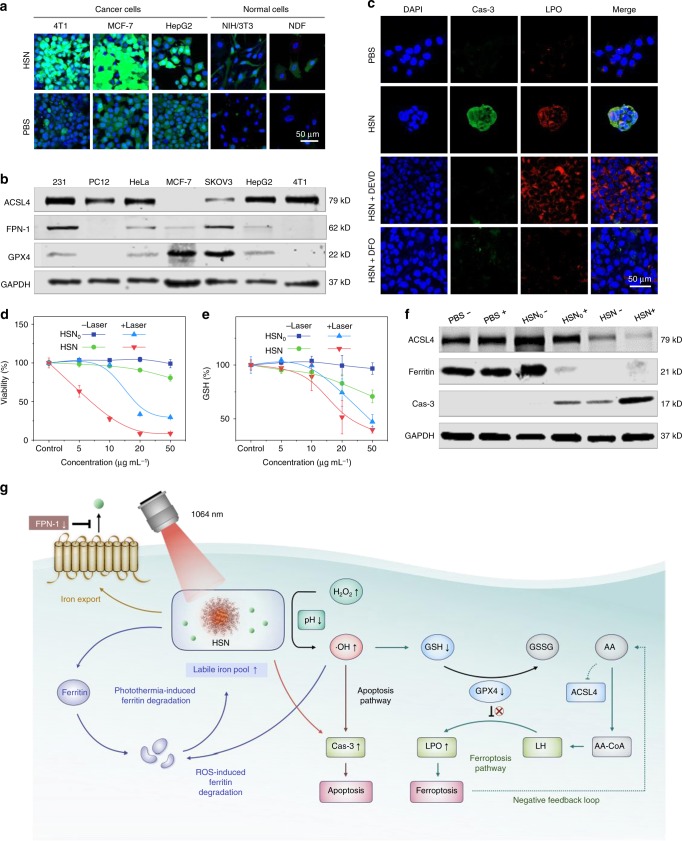
In vitro NIR-II photothermal ferrotherapy. **a** Confocal laser scanning microscopy (CLSM) images of different types of cancer cells and normal cells after incubation with HSN ([pTBCB] = 50 µg mL^−1^) or PBS for 24 h. ROS was indicated by green fluorescence from DCF-DA staining. Nuclei were stained with 4′,6-diamidine-2′-phenylindole dihydrochloride (DAPI) and indicated by blue fluorescence. 4T1: murine mammary carcinoma cell line; MCF-7: human breast adenocarcinoma cell line; HepG2: human hepatocellular carcinoma cell line; NIH/3T3: murine fibroblast cell line; NDF: normal human dermal fibroblast cell line. **b** Western immunoblot analysis of expression levels of ferroptosis-related proteins (ACSL4, FPN-1, GPX4) in a panel of cancer cell lines. MDA-MB-231 (231): human breast adenocarcinoma cell line; PC12: rat pheochromocytoma cell line; HeLa: human cervical adenocarcinoma cell line; SKOV3: human ovarian adenocarcinoma cell line. Source data were provided in Source Data File. **c** CLSM images of 4T1 cells after incubation with PBS, HSN, HSN with apoptosis inhibitor DEVD (100 µM), HSN with ferroptosis inhibitor deferoxamine (DFO) (100 µM) for 24 h, respectively. [pTBCB] = 50 µg mL^−1^. Expression of Cas-3 was indicated by immunofluorescence staining (green fluorescence), and lipid peroxidation was stained with a red-fluorescent probe BODIPY 665/676. Cell viabilities (**d**) and relative GSH levels (**e**) of 4T1 cells after incubation with HSN_0_ or HSN at various concentrations for 24 h with or without 1064 nm photoirradiation (1 W cm^−2^, 6 min). [pTBCB] = 50 µg mL^−1^. **f** Western immunoblots analysis of expression levels of ferroptosis and apoptosis related proteins in 4T1 cells in **d** and **e**. *Plus* and *minus* symbol indicated with and without photoirradiation, respectively. Source data were provided in Source Data File. **g** Proposed molecular mechanisms of HSN-mediated NIR-II photothermal ferrotherapy. GSSG glutathione disulfide, AA arachidonic acid, AA-CoA arachidonyl-CoA, LH phospholipid. Error bars indicated standard deviations of three independent measurements.

Catalytic activity of HSN was characterized against both cancer and normal cell lines. A fluorescent turn-on probe, 2′,7,′-dichlorofluorescin diacetate (DCF-DA), was used as the ROS indicator. After treatment with HSN, cancer cells showed much stronger green fluorescence than normal cells (Fig. 3a), revealing the catalytic specificity of HSN in cancerous cells due to their higher basal H_2_O_2_ level^43^. Because ferroptosis marked by lipid peroxidation (LPO) is known to have a vital role in ferrotherapy, ferroptosis sensitivity was screened over a panel of cancer cell lines through evaluation of expression levels of hallmark regulators^44,45^. They included: long-chain-fatty-acid-CoA ligase 4 (ACSL4), ferroportin-1 (FPN-1) and glutathione peroxidase 4 (GPX4). ACSL4, an enzyme that regulates arachidonic acid (AA) esterification to arachidonyl-CoA (AA-CoA) for phospholipid (LH) formation is a pivotal ferroptosis contributor^46^. FPN-1 is identified as the sole iron exporter of which the downregulation has been indicated to increase intracellular iron retention^47^. While GPX4 serves as a major ferroptosis suppressor by reducing phospholipid peroxides at the expense of glutathione (GSH)^48^. After western blotting analysis, 4T1 cells with upregulated ACSL4 yet downregulated FPN-1 and GPX4 levels were selected as the highly ferroptosis-sensitive cell line for subsequent studies (Fig. 3b).

In vitro therapeutic capability of HSN was investigated against 4T1 cells. After treating cells with HSN, cellular apoptosis was indicated by immunofluorescent staining (green fluorescence) of cleaved caspase-3 (Cas-3), whereas ferroptosis was indicated by LPO staining via a red-fluorescent probe BODIPY 665/676. As revealed in Fig. 3c, much stronger green and red fluorescence was observed in HSN-treated cells than control group, suggesting that endocytosed HSN triggered both apoptosis and ferroptosis in 4T1 cells. Further, addition of an apoptosis inhibitor (DEVD) ameliorated HSN-triggered apoptosis but had negligible effect on ferroptosis inhibition. However, both apoptosis and ferroptosis were inhibited after addition of a potent iron chelator deferoxamine (DFO), confirming that cell deaths were due to ferrous ions within HSN. Next, cell viabilities after in vitro cancer therapy were examined (Fig. 3d). In the absence of photoirradiation, HSN-mediated ferrotherapy caused slightly higher toxicity to 4T1 cells than the control treatment by HSN_0_ due to the catalytic activity of ferrous ion. With 1064 nm photoirradiation, HSN-mediated photothermal ferrotherapy induced the highest cytotoxicity among all treatments. For instance, at 50 µg mL^−1^, photothermal ferrotherapy induced a minimal cell viability of 8.7%, which was 3.4- and 9.3-fold lower than that for HSN_0_-mediated PTT (29.6%) or sole ferrotherapy (80.6%), respectively.

The underlying molecular mechanism of superior therapeutic efficacy of HSN-mediated photothermal ferrotherapy was studied. Intracellular GSH level as the representative of oxidative stress was measured by 5,5′-dithiobis(2-nitrobenzoic acid) (DTNB) assay after various treatments (Fig. 3e). A most significant drop of GSH level was observed in cells after photothermal ferrotherapy, followed by PTT or ferrotherapy. Consistently, flow cytometry analysis indicated the maximal ROS generation in 4T1 cells after photothermal ferrotherapy than sole PTT or ferrotherapy (Supplementary Fig. 11). Further, western blotting analysis indicated the most downregulated ACSL4 expression after NIR-II photothermal ferrotherapy (Fig. 3f), suggesting enhanced ferroptosis due to the presence of negative feedback loop possibly mediated by AA^49,50^. Besides, NIR-II photothermal ferrotherapy induced the highest Cas-3 expression, suggesting that cellular apoptosis was further enhanced. Because ferritin is the major intracellular iron storage protein, expression level of ferritin was also examined in cells after various treatments. Akin to ferrotherapy, photothermal ferrotherapy triggered more significant ferritin degradation than PTT, implying potentiated oxidative damage ascribed to the liberation of reactive iron from ferritin to replenish labile iron pool. The molecular mechanism of HSN-mediated photothermal ferrotherapy was summarized in Fig. 3g.

### In vivo NIR-II PA imaging-guided photothermal ferrotherapy

**Fig. 4:**
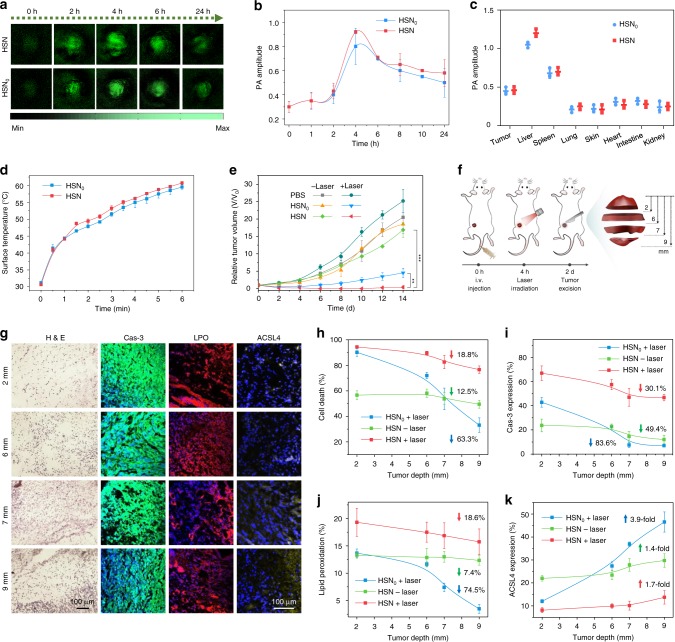
In vivo NIR-II PA imaging-guided photothermal ferrotherapy. **a** Time-course NIR-II PA images of tumor region on living mice bearing 4T1-xenograft tumor after intravenous administration of HSN or HSN_0_ ([pTBCB] = 250 µg mL^−1^, 200 µL per mouse, *n* = 3). Wavelength: 1064 nm. **b** Quantification of PA amplitudes in **a** (*n* = 3). **c** Biodistribution study of mice in **a** at 24 h post-administration of HSN or HSN_0_ (*n* = 3). **d** Surface tumor temperature of 4T1 tumor-bearing mice upon 1064 nm photoirradiation (1 W cm^−2^, 6 min) at 4 h after intravenous administration of HSN or HSN_0_ ([pTBCB] = 250 µg mL^−1^, 200 µL per mouse, *n* = 3). **e** Tumor growth curves of mice after photothermal ferrotherapy and monotherapies (*n* = 3). *P*-values were calculated by Student’s two-sided *t*-test. **P < 0.01, ***P < 0.001 (*n* = 3). **f** Scheme of photothermal depths of tumor. **g** H&E staining and immunofluorescent staining (Cas-3, LPO, and ACSL4) images of tumor sections at different photothermal depths after monotherapies or photothermal ferrotherapy. Cas-3, LPO, and ACSL4 staining was indicated with green, red, and yellow false colors, respectively. **h**–**k** Quantification of cell death percentage (**h**), Cas-3 expression (**i**), LPO extent (**j**), and ACSL4 expression (**k**) of tumor sections at different photothermal depths after monotherapies or photothermal ferrotherapy. *Upwards* or *downwards arrows* indicated the increased or decreased percentage at 9 mm relative to 2 mm. Error bars indicated standard deviations of three independent measurements.

To identify the optimal therapeutic window for in vivo therapy, NIR-II PA imaging was conducted on 4T1 tumor-bearing mice on a home-made PA system equipped with 1064 nm pulse laser. After systemic administration of HSN or HSN_0_, PA signals in tumor regions gradually increased and reached the maxima at 4 h post injection (Fig. 4a), suggesting the passive targeting of both nanoparticles in solid tumor probably through enhanced permeability and retention (EPR) effect due to their small hydrodynamic sizes and PEGylated surfaces (Fig. 2b, c). At this time point, the PA amplitude of tumor for HSN-treated mice was 3.1- and 1.2-fold higher than that of background and that for HSN_0_-treated mice (Fig. 4b), respectively. Such phenomenon should be mainly attributed to the superior PA property of HSN over HSN_0_ (Fig. 2f). Besides, ex vivo PA data at 24 h post injection revealed that the residual injected HSN or HSN_0_ mainly accumulated in liver, followed by spleen, tumor, and other organs (Fig. 4c).

Therapeutic potential of HSN-mediated NIR-II photothermal ferrotherapy was evaluated on 4T1 tumor-bearing mice and compared with monotherapies. According to PA imaging results, NIR-II photoirradiation was applied to tumor at 4 h post-administration of HSN or HSN_0_. Under photoirradiation, tumor temperatures for HSN and HSN_0_-treated mice gradually increased to 60.8 and 59.5 °C, respectively (Fig. 4d). After different treatments, tumor growth was monitored over time (Fig. 4e). Compared to PBS-treated group, mice treated with ferrotherapy showed minor retardation in tumor growth. Although PTT remarkably impeded tumor growth during the first few days, accelerated regrowth of tumor was observed 4 days after treatment. However, tumors treated with photothermal ferrotherapy were totally eradicated, and no sign of tumor regrowth was observed. Furthermore, negligible changes in body weights were found for mice during different treatments (Supplementary Fig. 15). And no significant physiological abnormalities could be found in hearts, spleens, and kidneys of living mice after either photothermal ferrotherapy or monotherapies (Supplementary Fig. 15), showing the acceptable biocompatibility of HSN and HSN_0_.

To unveil the mechanism of complete tumor elimination by HSN-mediated NIR-II photothermal ferrotherapy, tumors after various treatments were dissected at different depths (2, 6, 7, and 9 mm) in the direction of photoirradiation (termed as photothermal depth) (Fig. 4f). Hematoxylin and eosin (H&E) staining was performed followed by quantitative analysis (Fig. 4g, h, Supplementary Fig. 12). Overall, sole ferrotherapy displayed a relatively depth-independent therapeutic pattern (cell death decreased by 12.5% from 2 to 9 mm), whereas the therapeutic efficacy was inadequate (maximum 56.6% cell death). On the contrary, PTT demonstrated a depth-dependent therapeutic manner, showing largely compromised cell death with the increase of photothermal depth (cell death decreased by 63.3% from 2 to 9 mm) (Fig. 4h). And ablation depth in PTT was restricted to 6 mm. However, NIR-II photothermal ferrotherapy presented a relatively photothermal depth-independent therapeutic pattern akin to ferrotherapy (cell death only decreased by 18.8% from 2 to 9 mm), achieving efficient ablation at 9 mm that remarkably broke the record of reported therapeutic limitation in NIR-II window (~4 mm)^18^. Further, such ablation depth was almost comparable to the ablation radius (at centimeter-scale) of laser-induced thermal therapy, which relies on invasive surgery to directly deliver high-power laser into tumor interstitium for efficient ablation^51^.

At molecular levels, expression of apoptosis/ferroptosis biomarkers (Cas-3, LPO, and ACSL4) was evaluated at each photothermal depth after different treatments (Fig. 4g, i–k, Supplementary Fig. 13). Consistent with H&E results, NIR-II photothermal ferrotherapy displayed a photothermal depth-independent apoptosis/ferroptosis-inducing modality similar to ferrotherapy rather than PTT. For instance, from 2 to 9 mm, Cas-3 expression in photothermal ferrotherapy decreased by 30.1%, whereas that in PTT decreased by 83.6% and ferrotherapy decreased by 49.4%; LPO extent in photothermal ferrotherapy decreased by 18.6%, whereas that in PTT decreased by 74.5% and ferrotherapy merely decreased by 7.4%. Moreover, iron staining of tumor sections indicated evenly distributed iron stain in tumor tissue after photothermal ferrotherapy (Supplementary Fig. 14), in sharp comparison with ununiform bulky iron stains found after ferrotherapy. This was possibly because NIR-II photoirradiation potentiated self-degradation of HSN to tiny HSNS, which possessed a much favorable size (~1.7 nm) to escape high interstitial fluid pressure and small intratumoral cutoff pore for elevated convective permeation in tumor interstitium (Supplementary Fig. 10)^52^.

### Inhibition of lung and liver metastasis

**Fig. 5:**
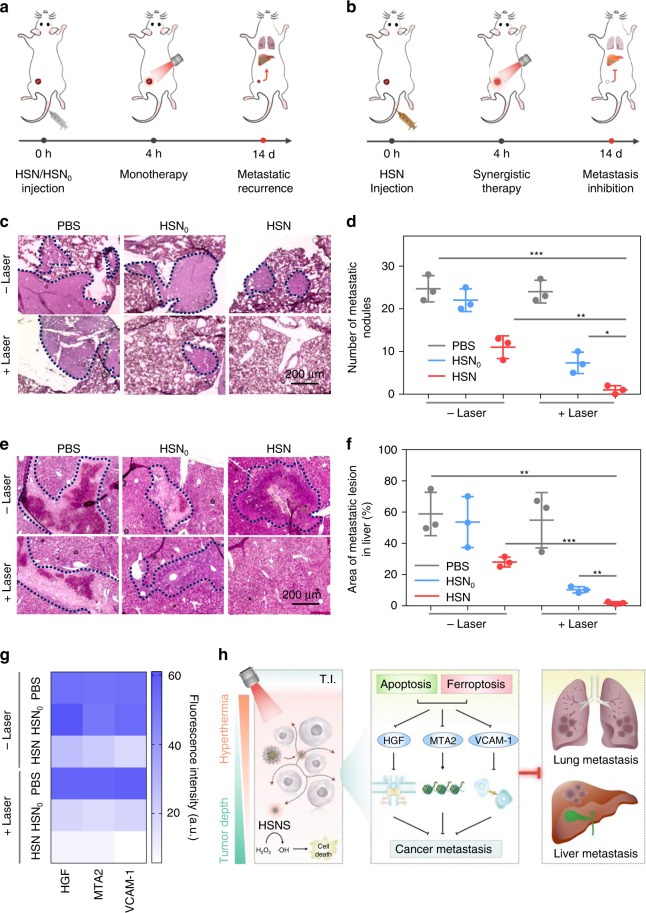
Cancer metastasis inhibition by HSN-mediated NIR-II photothermal ferrotherapy. **a**, **b** Schematic illustration of treatment schedule of monotherapies and photothermal ferrotherapy. H&E images of lung (**c**) and liver (**e**) metastasis of living mice after various treatments. Metastatic area was indicated by *dotted lines*. **d** Number of metastasis nodules per lung in mice after various treatments. **f** Area of metastatic lesions in liver per mice after various treatments. *P*-values were calculated by Student’s two-sided *t*-test. *P < 0.05, **P < 0.01, ***P < 0.001 (*n* = 3). **g** Mechanistic study and **h** illustration of molecular mechanism of metastasis inhibition by HSN-mediated NIR-II photothermal ferrotherapy. Quantification of expression levels of HGF, MTA2, and VCAM-1 in tumor tissues (at 9 mm, Supplementary Fig. 16) in living mice. T.I. tumor interstitium. Error bars indicated standard deviations of three independent measurements.

Given that distant metastasis is widely observed in clinical settings of breast cancer, anti-metastasis performance of HSN-mediated NIR-II photothermal ferrotherapy was evaluated and compared with monotherapies (Fig. 5a, b). After 14 days of different treatments, pulmonary metastases of living mice were examined using H&E staining. Fewer pulmonary metastatic nodules were counted in PTT or ferrotherapy-treated mice than PBS-treated group, whereas hardly any nodule could be found in photothermal ferrotherapy-treated mice (Fig. 5c, d). Further, metastatic status in liver was examined (Fig. 5e, f). Consistent with the trend of pulmonary metastasis, minor extent of hepatic metastatic lesions was observed in monotherapy-treated mice than control group; whereas almost no noticeable hepatic metastatic lesions were found in photothermal ferrotherapy-treated mice. Together, these data emphasized that HSN-mediated photothermal ferrotherapy almost completely arrested distant cancer metastasis, which was not possible for either monotherapy.

To unveil the molecular mechanism of efficient metastasis inhibition by HSN-mediated NIR-II photothermal ferrotherapy, expression levels of metastasis-related proteins in tumor were evaluated after different treatments (Supplementary Fig. 13). These proteins included: hepatocyte growth factor/scatter factor (HGF), which activates tyrosine kinase Met signaling to promote cancer cell growth and motility for metastatic spread^53^; metastasis-associated protein 2 (MTA2), which is a crucial transcriptional repressor in nuclear remodeling, and deacetylation (NuRD) complex to regulate cytoskeletal and motility pathways for metastatic dissemination^54^; and vascular cell adhesion molecule-1 (VCAM-1) of which the specific binding to very late antigen 4 (VLA-4)-expressing leukocytes is responsible for metastatic invasion of malignant breast cancer cell to distant organs^55^. Immunofluorescence staining was performed to tumor sections followed by quantitative analysis. Compared with PBS-treated tumors (Supplementary Fig. 16), much weaker green fluorescence assigned to HGF, MTA2 and VCAM-1 staining could be found in tumors after monotherapies. However, green fluorescence could be hardly observed in tumors after photothermal ferrotherapy, showing the most downregulated expression of these proteins. Further, quantitative analysis indicated that HGF expression in photothermal ferrotherapy dropped to 35 and 49% of that in ferrotherapy and PTT, respectively. Similarly, MTA2 expression dropped to 40 and 54%; and VCAM-1 expression dropped to 31 and 29% (Fig. 5g). Together, molecular mechanism of photothermal ferrotherapy-mediated metastasis inhibition was summarized in Fig. 5h.

## Discussion

We synthesized the first semiconducting nanozyme (HSN) based on the complexation of an organic semiconducting polymer (pTBCB-PEG) and Fe^2+^ for NIR-II PA imaging-guided photothermal ferrotherapy (Fig. 1a). Different from ordinary iron delivery systems (such as phenolic acid^7,21^, oxidized starch^56^, leukocyte membrane^8^, etc.) that have no optical properties, pTBCB-PEG possessed extremely narrow band gaps for efficient photothermal transduction in the NIR-II window (Fig. 2a). Probably because of iron chelation-enhanced intramolecular interactions (Supplementary Fig. 8), HSN demonstrated almost the highest PCE (98.9%) among all the reported NIR-II photothermal agents. Under NIR-II photoirradiation, such excellent photothermal performance of HSN enabled improved PA delineation of tumor, enhanced Fenton reaction to elevate ·OH production (Fig. 2g) and promoted self-degradation to tiny fragments for increased intratumoral penetration.

Molecular mechanism of NIR-II photothermal ferrotherapy was unveiled using HSN as the model system (Fig. 3). Because of the upregulated expression of ACSL4 yet downregulated level of and FPN-1 and GPX4, 4T1 cell line demonstrated high ferroptosis sensitivity and was utilized for anti-cancer study. Under NIR-II photoirradiation, HSN-mediated photothermal heating, which (i) triggered severe cellular apoptosis, (ii) promoted the degradation of ferritin to further replenish labile iron pool, (iii) amplified the catalytic activity of HSN/HSNS to promote ·OH production for enhanced apoptosis/ferroptosis to cause overwhelming cell death. Thereby, HSN-mediated NIR-II photothermal ferrotherapy augmented the therapeutic effect of monotherapies via a sophisticated molecular network involving interlaced synergism of both programmed cell death modalities.

Despite the recent progress in NIR-II PTT, inadequate photothermal ablation in deep tumor due to exponential attenuation of photons greatly limits its clinical applications. The maximum therapeutic depth was restricted to 4 mm in bulky solid tumor via intravenous administration of 2D niobium carbide^18^, or to 5 mm through intratumoral injection of the reported SPN^34^. However, HSN-mediated NIR-II photothermal ferrotherapy presented a relatively photothermal depth-independent therapeutic paradigm, realizing tumor elimination at an unexplored depth of 9 mm. Such superior therapeutic depth was attributed to the seamless synergy of PTT and ferrotherapy: (i) HSN-mediated photoinduced hyperthermia and photothermally enhanced Fenton reaction; (ii) elevated intratumoral diffusion induced by self-degradation of HSN. Such deep-tissue eradication effectively impeded tumor regrowth, preventing distant metastasis to lung and liver. At cellular level, mechanistic study uncovered that HSN-mediated NIR-II photothermal ferrotherapy downregulated an array of metastasis-related proteins.

In summary, we reported a hybrid polymeric nanozyme (HSN) that compensates the limitations of both ferrotherapy and PTT for NIR-II PA imaging-guided combination cancer therapy. To the best of our knowledge, our study not only pushes the therapeutic depth of non-invasive PTT to an unprecedent level, but also uncovers the biological mechanism of photothermal ferrotherapy. More broadly, the nanozyme strategy presented here can be generalized to develop photothermal ferro-therapeutic agents for other deep-tissue-seated diseases such as neurodegenerative diseases.

## Methods

### Chemicals

All the chemicals were purchased from Sigma-Aldrich or Tokyo Chemical Industry unless otherwise stated. 2,6-Dibromo-4H-cyclopenta[2,1-b:3,4-b′]dithiophene and 2,6-bis(trimethyltin)-4,8-didodecylbenzo[1,2-b;4,5-b′]dithiophene were purchased from Luminescence Technology Corp (Lumtec). 4,9-Dibromo-6,7(4-hexylphenyl)-[1,2,5]thiadiazolo[3,4-g]quinoxaline was purchased from Brilliant Matters. Secondary antibodies for immunoblotting, which include IRDye 800 CW goat anti-mouse (1:10,000; 925-32210) and IRDye 680 CW goat anti-rabbit (1:10,000; 925-68071) were purchased from LI-COR Biosciences. Anti-HGF (1:500; ab83760), anti-MTA2 (1:100; ab8106), anti-VCAM-1 (1:250; ab134047) antibodies were purchased from Abcam. Secondary antibody Alexa Fluor 488 conjugated goat anti-rabbit IgG H&L (1:500; ab150077) for immunofluorescent staining was purchased from Abcam.

### Material characterization

Absorption spectra were measured on a Lambda 950 spectrometer. Proton nuclear magnetic resonance (^1^H NMR) spectra were recorded on a Bruker Avance II 300 MHz NMR spectrometer. Fluorescence spectra were measured on a Fluorolog 3-TCSPC spectrofluorometer (Horiba Jobin Yvon). Dynamic light scattering and zeta potential were recorded on a Malvern Nano-ZS Particle Sizer. Transmission electron microscope (TEM) images were captured on a JEOL JEM 1400 transmission microscope. Scanning transmission electron microscopy-energy dispersive X-ray (STEM-EDX) element mapping was performed on JEOL 2100F with ultra-high resolution (UHR) configuration (accelerating voltage: 200 kV). Solution temperature for photostability study was measured by a FLIR T420 thermal camera. Gel permeation chromatography was performed on a Shimazu LC-VP system (standard: styrene; eluent: tetrahydrofuran). Confocal images were captured on Zeiss LSM800. Flowcytometry was performed on Fortessa X20 (BD Biosciences). Western blot images were captured on Licor Odyssey CLx fluorescence imaging system. 1064 nm laser was purchased from Shanghai Connet Fiber Optics Co., Ltd. (Shanghai, China).

### Synthesis of monomer 1

2,6-Dibromo-4H-cyclopenta[2,1-b:3,4-b′]dithiophene (1 g, 3 mmol), 50% NaOH solution (freshly prepared, 64 mL) and tetrabutylammonium bromide (TBAB, 200 mg) were weighed and placed in a 250 mL round-bottom flask. The mixture was then degassed via freeze–thaw cycles, followed by reflux at 75 °C for 20 min under the protection of N_2_ atmosphere. 1,6-Dibromohexane (3 mL, 7.5 mmol) was then dropwise added to the flask via a syringe and the reaction was allowed to carry out for 80 min at 75 °C. After reaction, the mixture was cooled down to room temperature, extracted with ethyl acetate, washed three times with water, and dried with anhydrous sodium sulfate. Thereafter, the crude monomer 1 was concentrated via rotary evaporation followed by purification via silica gel column chromatography using hexane as the eluent. ^1^H NMR (300 MHz, CDCl_3_) *δ*: 6.93 (s, 2H), 3.34 (*t*, 4H), 1.74 (m, 8H), 1.34–1.26 (m, 4H), 1.19–1.09 (m, 4H), 0.92–0.84 (m, 4H).

### Synthesis of pTBCB-PEG

To synthesize pTBCB-Br, 2,6-bis(trimethyltin)-4,8-didodecylbenzo[1,2-b;4,5-b′]dithiophene (32 mg, 0.0375 mmol), 4,9-dibromo-6,7(4-hexylphenyl)-[1,2,5]thiadiazolo[3,4-g]quinoxaline (20 mg, 0.03 mmol), monomer 1 (4.74 mg, 0.0075 mmol), Pd_2_(dba)_3_ (1.2 mg, 0.0012 mmol), and tri(*o*-tolyl)phosphine (3.6 mg, 0.0108 mmol) were weighed and placed in a 50 mL Schlenk flask. After addition of cholorobenzene (4 mL), the mixture was degassed by three freeze–pump–thaw cycles. Thereafter, the reaction was carried out at 100 °C under the protection of nitrogen atmosphere for 4 h. After reaction, the mixture was cooled down to room temperature, followed by dropwise addition to cold methanol. After vigourous stirring, the dark precipitates in methanol was collected via centrifugation, washed three times with cold methanol to remove impurities, and then dried under vacuum to afford dark solids of pTBCB-Br.

To synthesize pTBCB-N_3_, pTBCB-Br (23 mg) and sodium azide (20 mg) was dissolved in a mixture (12 mL) of tetrahydrofuran (THF) and *N*,*N*-dimethylformamide (DMF) (THF:DMF 2:1). After stirring at room temperature for 24 h, the mixture was concentrated by rotary evaporation, re-dissolved in dichloromethane, washed three times with water, and dried over anhydrous sodium sulfate. The obtained pTBCB-N_3_ (23 mg) and methoxypolyethylene glycol dibenzocyclooctyne (DBCO-mPEG2000) (100 mg) were dissolved in THF (6 mL) and stirred at room temperature for 24 h. After reaction, the solvent was removed by rotary evaporation and the obtained solids were re-dissolved in deionized water. Thereafter, the excess DBCO-mPEG2000 was removed by ultracentrifugation (MWCO 50 kDa) at 5780×*g* at 4 °C. After purification, the concentrated aqueous solution was lyophilized to obtain pTBCB-PEG powder. ^1^H NMR (300 MHz, CDCl_3_) *δ*: 8.10-7.30 (m, 8H), 7.26–6.78 (m, 4H), 4.04–3.81 (m, 2H), 3.78–3.53 (m, 72H), 3.51–3.25 (m, 4H), 2.08–1.86 (m, 10H), 1.73–1.63 (m, 4H), 1.57–1.09 (m, 48H), 0.94–0.78 (m, 12H).

### Preparation of HSN_0_ and HSN

To prepare HSN_0_, lyophilized pTBCB-PEG powder (1 mg) was dissolved in THF (1 mL), followed by rapid injection into deionized water (8 mL) under vigorous sonication. Excess THF was then removed under a gentle N_2_ flow, and the remaining aqueous solution was filtered through a 220 nm polyvinylidene fluoride (PVDF) syringe-driven filter (Millipore), followed by concentration via ultracentrifugation at 5780×*g* for 30 min at 4 °C to afford stock solution.

As for the preparation of HSN, freshly prepared ferrous sulfate solution (0.1 M, 0.5 mL, pH 4.0) was dropwise added to HSN_0_ solution (300 µg mL^−1^, 2 mL) under vigorous stirring. After continuous stirring overnight, the excess ferrous ion was removed by three cycles of ultracentrifugation at 3260×*g* for 25 min at 4 °C followed by water rinse. The purified HSN solution was then diluted and filtered via a 220 nm PVDF syringe-driven filter and ultra-centrifuged to afford HSN stock solution.

### In vitro photothermal study

To measure photostability, HSN_0_ and HSN solution (12 µg mL^−1^, 200 µL) was irradiated with 1064 nm laser (1 W cm^−2^) for 6 min followed by natural cooling for another 6 min after removal of photoirradiation. Five cycles of heating-cooling process were carried out and solution temperature was monitored by IR thermal camera.

### Calculation of photothermal conversion efficiency

Photothermal conversion efficiency was measured and calculated according to literature^15^. HSN or HSN_0_ solution (2 mL, optical density at 1064 nm = 1) was placed in a 3.5-mL quartz cuvette (5.66 g, Sangon Biotech, Shanghai, China), followed by 1064 nm laser irradiation (1 W cm^−2^) for 30 min to reach the thermal equilibrium and subsequent natural cooling process in the absence of laser irradiation. During the measurement, solution temperature was monitored by a dual input J/K type thermometer (TM300, Extech Instruments, Waltham, MA). Calculation was briefly given as follows:

Energy input and dissipation in the measurement system could be expressed as:

1 $${\mathop {\sum}\limits_i} {m_i} C_{p,i}\frac{{{\mathrm{d}}T}}{{{\mathrm{d}}t}} = Q_{{\mathrm{NP}}} + Q_{{\mathrm{sys}}} - Q_{{\mathrm{diss}}},$$

where *m*_*i*_ and *C*_*p,i*_ are the mass and heat capacity of the component (e.g. water and quartz cuvette) in the measurement system, respectively. *Q*_NP_ stands for the input of energy from HSN or HSN_0_; *Q*_sys_ is the energy input from the other components in the measurement system; and *Q*_diss_ represents the loss of energy from measurement system to surroundings. The laser-induced term *Q*_NP_ could be interpreted as:

2 $$Q_{{\mathrm{NP}}} = I(1 - 10^{ - {{A}}_\lambda })\eta,$$

where *I* is the power of 1064 nm laser; *A*_*λ*_ is the absorbance of HSN or HSN_0_ at 1064 nm; *η* stands for photothermal conversion efficiency. On the other hand, *Q*_diss_ could be expressed as:

3 $$Q_{{\mathrm{diss}}} = hS(T - T_{{\mathrm{surr}}}),$$

where *h* is heat transfer coefficient; *S* is surface area of the quartz cuvette exposed to laser; *T*_surr_ is the ambient temperature. When the measurement system reached a thermal equilibrium, temperature was recorded as *T*_max_. At this time, the total energy input to the system is equal to the energy dissipation:

4 $$Q_{{\mathrm{NP}}} + Q_{{\mathrm{sys}}} = Q_{{\mathrm{diss}}} = hS(T_{\max } - T_{{\mathrm{surr}}}).$$

After removal of laser irradiation, the energy input drops to zero, and Eq. 1 is expressed as:

5 $${\mathop {\sum}\limits_i} {m_i} C_{p,i}\frac{{{\mathrm{d}}T}}{{{\mathrm{d}}t}} = - {{Q}}_{{\mathrm{diss}}} = - hS(T - T_{{\mathrm{surr}}}).$$

After rearrangement followed by integration, Eq. 5 gives:

6 $$t = - \frac{{{\mathop {\sum}\limits_i }{m_i} C_{p,i}}}{{hS}}\ln \frac{{T - T_{{\mathrm{surr}}}}}{{T_{\max } - T_{{\mathrm{surr}}}}}.$$

The time constant *τ*_s_ is expressed as:

7 $$\tau _{\mathrm{s}} = \frac{{{\mathop {\sum}\limits_i} {m_i} C_{p,i}}}{{hS}}.$$

A dimensionless factor *θ* is defined as:

8 $$\theta = \frac{{T - T_{{\mathrm{surr}}}}}{{T_{\max } - T_{{\mathrm{surr}}}}}.$$

Then Eq. 6 could be expressed as:

9 $$t = - \tau _{\mathrm{s}}\ln \theta.$$

Therefore, *τ*_s_ could be calculated by linear regression of time versus negative ln*θ*. And *hS* could be obtained through Eq. 7. *Q*_sys_ could be measured by replacing nanoparticles with pure solvent:

10 $$Q_{{\mathrm{sys}}} = hS(T_{\max ,\,{\mathrm{H}}_{\mathrm{2}}{\mathrm{O}}} - T_{{\mathrm{surr}}}).$$

At last, *η* could be calculated as:

11 $$\eta = \frac{{hS\left( {T_{\max } - T_{{\mathrm{surr}}}} \right) - Q_{{\mathrm{sys}}}}}{{I(1 - 10^{ - {{A}}_\lambda })}}.$$

### Cell culture and ROS detection

4T1 murine mammary carcinoma cell line, MCF-7 human breast adenocarcinoma cell line, HepG2 human hepatocellular carcinoma cell line, NIH/3T3 murine fibroblast cell line, NDF normal human dermal fibroblast cell line, MDA-MB-231 human breast adenocarcinoma cell line, PC12 rat pheochromocytoma cell line, HeLa human cervical adenocarcinoma cell line, and SKOV3 human ovarian adenocarcinoma cell line (purchased from American Type Culture Collection, ATCC) were cultured in Dulbecco’s Modified Eagle Medium (DMEM) supplemented with 10% fetal bovine serum (FBS) and 1% antibiotics (penicillin and streptomycin) and placed in a humid CO_2_ incubator providing an atmosphere containing 5% CO_2_ at 37 °C.

To detect the intracellular ROS aroused by HSN, cells were seeded in confocal cell culture dishes at a density of 1 × 10^4^ cells per dish. After culture in incubator for 12 h, HSN (final concentration: [pTBCB] = 50 µg mL^−1^) or PBS were added to the cells and incubated with cells for 24 h. Thereafter, cells were gently washed three times with fresh PBS to remove excess nanoparticles, and then fixed with 4% paraformaldehyde. After fixation, cells were successively stained with DAPI and DCF-DA. Then cells were imaged under a LSM800 confocal microscope.

### Western blotting

Cells were lysed via vigorous sonication on ice bath and protein concentration was determined by Bradford protein assay. Thereafter, immunoblotting of cell lysates was performed using antibodies to ACSL4 (1:1000; ab205199; Abcam), FPN-1 (1:1000; NBP1-21502SS; Singlab Technologies Pte Ltd), GPX4 (1:100; sc-166120; Axil Scientific Pte Ltd), cleaved cas-3 (1:500; 9661L; Cell Signaling Technology), ferritin (1:500; MA532244; Life Technologies Holdings Pte Ltd), and GAPDH (1:500; sc-32233; Axil Scientific Pte Ltd) according to standard protocols. Source data of scans of blots were provided in Source Data File.

### In vitro examination of apoptosis and ferroptosis

4T1 cells were seeded in confocal cell culture dishes at a density of 1 × 10^4^ cells per dish. After culture in incubator for 12 h, HSN (final concentration: [pTBCB] = 50 µg mL^−1^), HSN with DEVD (100 µM), HSN with DFO (100 µM), and PBS were, respectively, added to the cells. After incubation for 24 h, cells were gently washed three times with fresh PBS and then fixed with 4% paraformaldehyde. After fixation, cells were incubated with PBS containing 0.1% Triton X-100 (PBST) and then washed three times with ice-cold PBS. Afterwards, cells were incubated with 3% bovine serum albumin (BSA) in PBST for 30 min. Then cells were washed three times in PBS and incubated with primary cleaved caspase-3 antibody (Cell Signaling Technology) (1:1000 in PBST) in a humidified chamber at 4 °C overnight. After incubation, cells were washed three times in PBS and incubated with secondary antibody Alexa Fluor 488 conjugated donkey anti-rabbit IgG (Thermo Fisher Scientific) (1:1000 in PBST) at room temperature for 1 h. Then cells were washed with PBS and sequentially stained with BODIPY 665/676 (Thermo Fisher Scientific) (10 µM, 30 min) and DAPI. After mounted by Fluoromount aqueous mounting medium, cells were imaged under a LSM800 confocal microscope.

### In vitro cancer therapy

4T1 cells were seeded in 96-well plates (1 × 10^4^ cells per well) and cultured in incubator for 24 h. Then cells were treated with HSN_0_ or HSN at different concentrations (0, 5, 10, 20, 50 µg mL^−1^). After incubation for 24 h, cells were treated with or without 1064 nm laser irradiation (1 W cm^−2^, 6 min). After incubation for another 24 h, cells were gently washed three times with fresh PBS. Thereafter, 120 μL freshly prepared working solution (containing 100 µL DMEM and 20 μL MTS reagent) was added to each well. After incubation for 3 h, absorbance at 490 nm was measured on a SpectraMax M5 microplate reader. And cell viability was determined as the ratio of the absorbance of cells with nanoparticle and/or laser treatment to that of blank cells without any treatments.

### DTNB assay and DCF-DA assay

DTNB assay was utilized to measure GSH level of 4T1 cells in in vitro cancer therapy. After various treatments of 4T1 cells, cells were gently washed three time with fresh ice-cold PBS, followed by sonication in ice bath to afford cell lysate. Thereafter, Ellman’s reagent (DTNB) was added to cell lysate (final concentration: 200 µM) and incubated at room temperature for 30 min. Later, the absorbance at 412 nm was measured on a SpectraMax M5 microplate reader and the relative level of GSH was determined by the ratio of absorbance for experimental group to that for control group (blank cells without any treatment).

DCF-DA assay was utilized to measure intracellular ROS level of 4T1 cells in in vitro cancer therapy. After various treatment of 4T1 cells, cells were gently washed three time with fresh PBS and incubated with DCFH-DA reagent. Then, cells were washed three times in ice-cold PBS, trypsinized, and resuspended in fresh PBS for flow cytometry test.

### Tumor mouse model

Animal experiments in Singapore were performed in compliance with Guidelines for Care and Use of Laboratory Animals of the Nanyang Technological University-Institutional Animal Care and Use Committee (NTU-IACUC) and approved by the Institutional Animal Care and Use Committee (IACUC) for Animal Experiment, Singapore. Animal experiments in China were performed in strict accordance with the NIH guidelines for the care and use of laboratory animals (NIH Publication No. 85-23 Rev. 1985) and approved by the Institutional Animal Use and Care Committee of Shan Xi Medical University (Approval No. 2016LL141, Taiyuan, China). Two million 4T1 cells suspended in 0.2 mL supplemented DMEM were subcutaneously injected into the right flank of the female NCr nude mice (6-week-old). Tumors were allowed to grow for 2 weeks before in vivo cancer therapy.

### NIR-II PA tomography system

PA characterization was conducted on a home-made NIR-II PA tomography system. A 1064 nm Nd:YAG pulse (5 ns, 10 Hz) laser (Continuum, Surelite Ex) was utilized as excitation source. Briefly, the 1064 nm beam was directed to the single-ultrasound transducer (UST) (V323-SU/2.25 MHz, Olympus NDT) scanner, and expanded by an optical diffuser to the inspection area. Water was utilized as the medium and UST was immersed in water so as to couple the acoustic signal to the transducer. The collected PA signals were then amplified and band-pass filtered (1–10 MHz) by ultrasound receiver unit (Olympus NDT, 5072PR). Later, PA signals were subsequently transmitted to a computer with a data acquisition card (25 Ms/s, GaGe, compuscope 4227) for digitalization and recording. PA images of samples or mice were reconstructed via a delay-and-sum back projection algorithm.

For in vivo PA imaging, NCr nude mice bearing 4T1-xenograft tumor were anesthetized and administered with HSN or HSN_0_ ([pTBCB] = 250 µg mL^−1^, 200 µL per mouse, *n* = 3) via tail vein injection. PA images were captured at designated time points before and after sample administration.

### Ex vivo biodistribution

At 24 h post injection of HSN or HSN_0_, mice were killed. Tumors, livers, spleens, lungs, skin, hearts, intestines, and kidneys were harvested and embedded in 1% agar gel phantom. PA signals from embedded organs were measured on the home-made PA tomography system.

### In vivo anti-cancer therapy

4T1 tumor-bearing mice were intravenously injected with PBS (200 μL), HSN_0_, or HSN ([pTBCB] = 250 μg mL^−1^, 200 μL per mouse). At 4 h post injection, tumors on mice for PTT and synergistic therapy were irradiated with 1064 nm laser at 1 W cm^−2^ for 6 min. Tumor surface temperature was monitored by an IR thermal camera throughout irradiation process. Afterwards, tumor sizes and body weights of mice were monitored every two days for 14 days. The tumor volume was expressed as follows:

$${{V}} = \left( {{\mathrm{Tumor}}\,{\mathrm{length}}} \right) \times \left( {{\mathrm{tumor}}\,{\mathrm{width}}} \right)^2/2.$$

Thus, relative tumor volume was calculated as *V*/*V*_0_ (*V*_0_ indicated the initial tumor volume).

### Histological studies, immunofluorescence, and iron staining

After 2 days of in vivo anti-cancer therapy, mice were killed, and tumors were excised for histological examination. Briefly, harvested tumors were fixed in 4% paraformaldehyde, followed by paraffin-embedding according to standard protocol and dissection to 10-µm tissue sections at different depths (2, 6, 7, 9 mm) in the direction of laser irradiation (photothermal depth). Then tissue sections were stained with H&E according to standard protocol and imaged under a Nikon ECLIPSE 80i microscope. Area of cell death was quantified using ImageJ.

As for immunofluorescent staining, the fixed tumors were dehydrated overnight in 30% sucrose, embedded in optimal cutting temperature (O.C.T.) medium, and sectioned into 10 µm-slices on a cryostat (Leica, CM1950) at different photothermal depths (2, 6, 7, 9 mm). Thereafter, immunofluorescent staining of cas-3 and ACSL4 as well as LPO staining was performed following the same procedure as the above-described apoptosis/ferroptosis staining. For iron staining, cryostat tissue sections at each photothermal depths (2, 6, 7, 9 mm) were stained according to standard Prussian blue stain protocol and then imaged under LX71 inverted microscope (Olympus).

### Ex vivo lung and liver metastasis examination

After 14 days of in vivo anti-cancer therapy, 4T1 tumor-bearing mice were killed and major organs including hearts, livers, spleens, lungs, and kidneys were harvested. Metastatic nodules in lungs were counted. Thereafter, organs were fixed in 4% paraformaldehyde, embedded in paraffin, sectioned to thin slices (10 µm), and stained with H&E according to standard protocols. Organ sections were then imaged under Nikon ECLIPSE 80i microscope and metastatic area in livers were quantified using ImageJ. Immunofluorescent staining of metastasis-related proteins was performed according to the above-described procedure.

### Data analysis

Results of experiments were presented as mean ± standard deviation unless otherwise stated. PA signals were analyzed using MATLAB. Statistical differences between two groups were calculated by two-tailed Student’s *t*-test using GraphPad Prism 7 (GraphPad Software, Inc., CA, USA). For all statistical analysis, **P < 0.05 was regarded as statistically significant.

### Reporting summary

Further information on research design is available in the Nature Research Reporting Summary linked to this article.

## Supplementary information

Supplementary information

Reporting Summary

## Data Availability

All data needed to evaluate the conclusions in the paper are presented in the paper and/or the Supplementary Materials. Additional data related to this paper may be requested from the authors.
