# Supplementary material for: Transformable hybrid semiconducting polymer nanozyme for second near-infrared photothermal ferrotherapy
Source: Nat Commun. 2020 Apr 20;11:1857. doi: 10.1038/s41467-020-15730-x (PMC7170847; doi:10.1038/s41467-020-15730-x)
Supplement: Supplementary file 1 — Supplementary information [file 41467_2020_15730_MOESM1_ESM.docx]

Supplementary Materials for

**Hybrid semiconducting polymer nanozyme for second near-infrared photothermal ferrotherapy**

*Yuyan Jiang^1^,* Xuhui Zhao*^2^, Jiaguo Huang^1^, Jingchao Li^1^, Paul Kumar Upputuri^1^, He Sun^3^, Xiao Han^3^, Manojit Pramanik^1^, Yansong Miao^3^, Hongwei Duan^1^, Kanyi Pu^1,^*, and Ruiping Zhang^2,^**

^1^School of Chemical and Biomedical Engineering, Nanyang Technological University, 70 Nanyang Drive, Singapore 637457, Singapore.

^2^The Affiliated Da Yi Hospital of Shanxi Medical University, Taiyuan, Shanxi, 030032, P. R. China

^3^School of Biological Science, Nanyang Technological University, Singapore 637551, Singapore.

(email: [zrp_7142@sxmu.edu.cn](mailto:zrp_7142@sxmu.edu.cn); kypu@ntu.edu.sg)


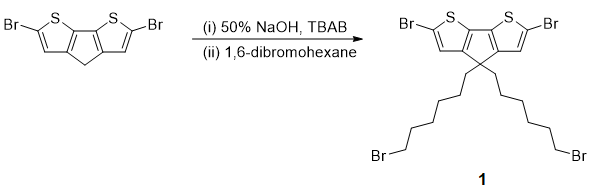


**Supplementary Figure 1.** Synthetic route of monomer 1. TBAB, tetrabutylammonium bromide.





**Supplementary Figure 2.** Synthetic route of pTBCB-PEG. Pd_2_(dba)_3_, tris(dibenzylideneacetone)- dipalladium(0). DBCO-mPEG2000, methoxypolyethylene glycol dibenzocyclooctyne, Mn = 2 kDa.


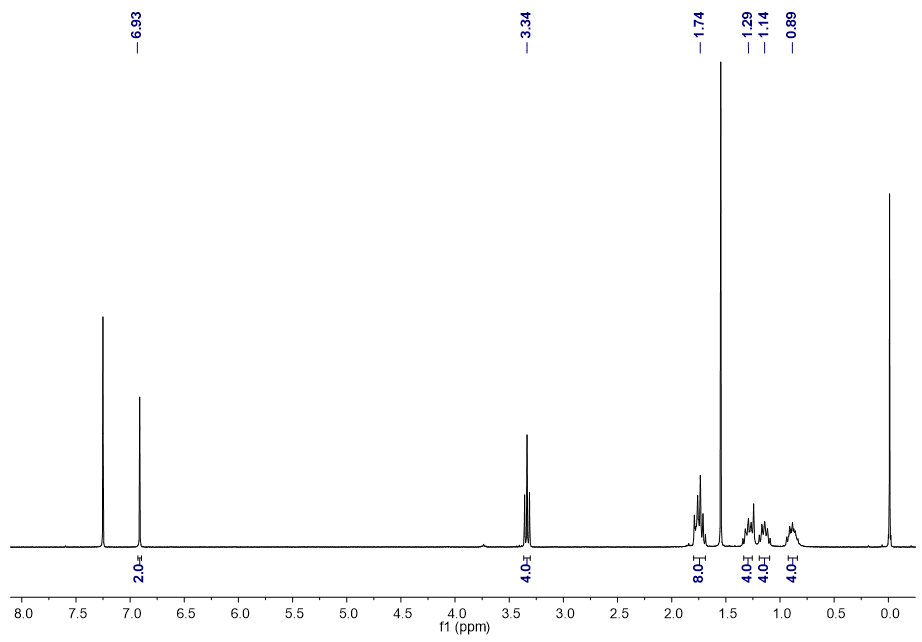


**Supplementary Figure 3.** ^1^H NMR spectrum of monomer 1 in CDCl_3_.


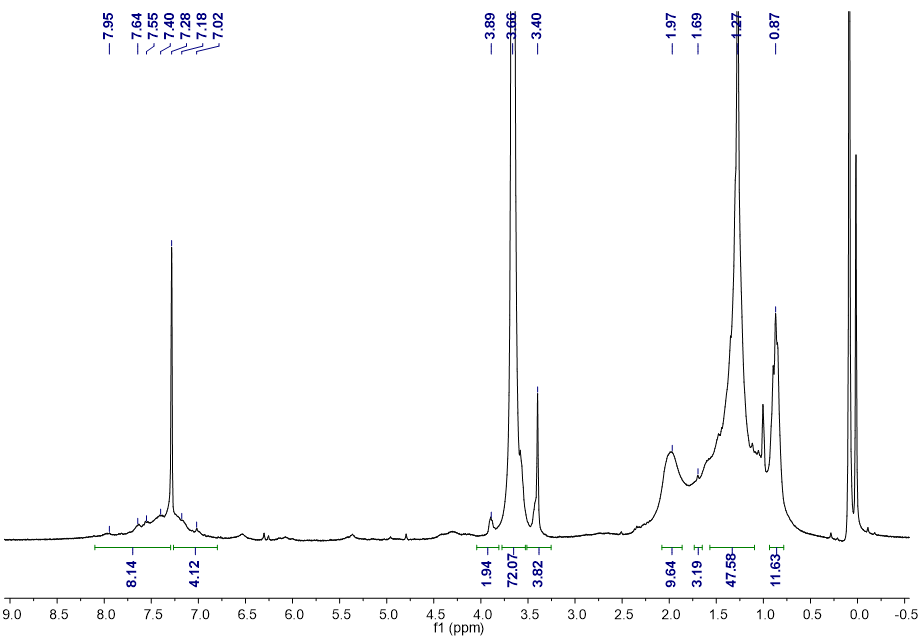


**Supplementary Figure 4.** ^1^H NMR spectrum of pTBCB-PEG in CDCl_3_.

**Supplementary Figure 5.** Schematic illustration of HSN_0_.


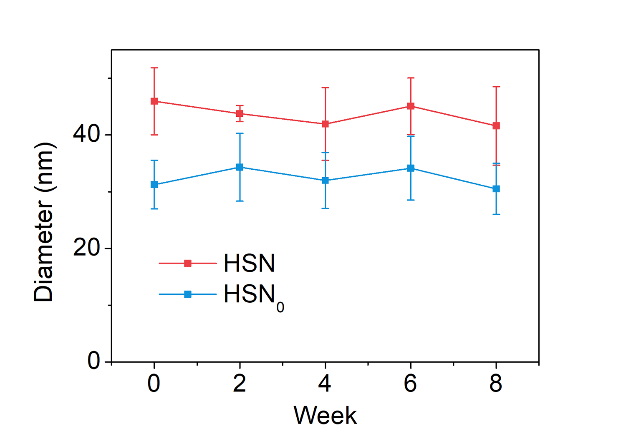


**Supplementary Figure 6.** DLS profiles of HSN and HSN_0_ during storage in 1 × PBS.


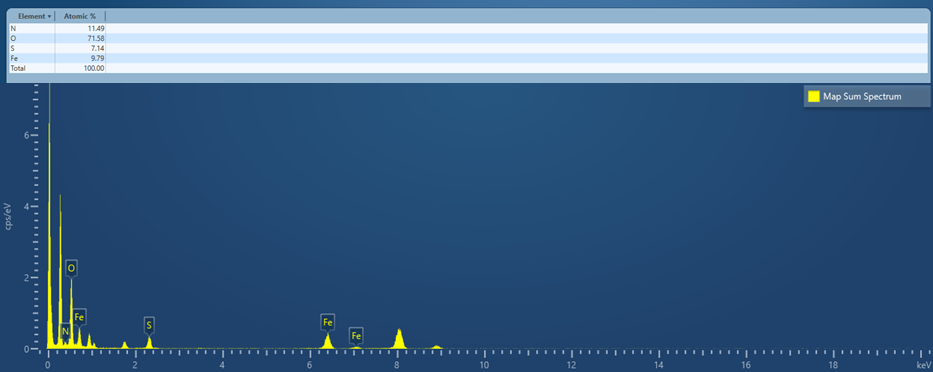


**Supplementary Figure 7.** STEM-EDX element analysis of HSN.


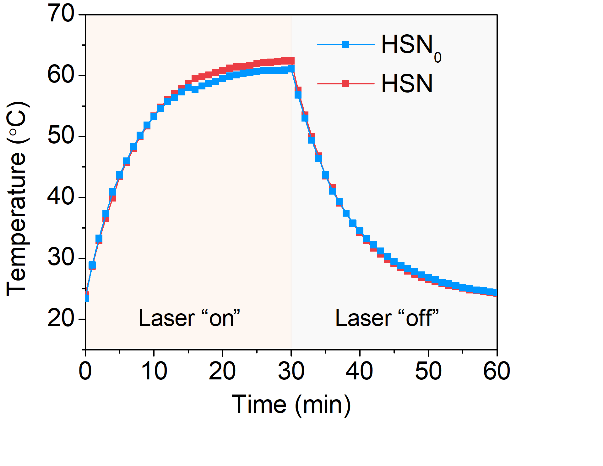


**Supplementary Figure 8.** Measurement of photothermal conversion efficiency of HSN_0_ and HSN at 1064 nm (1 W/cm^2^). Optical density at 1064 nm = 1.


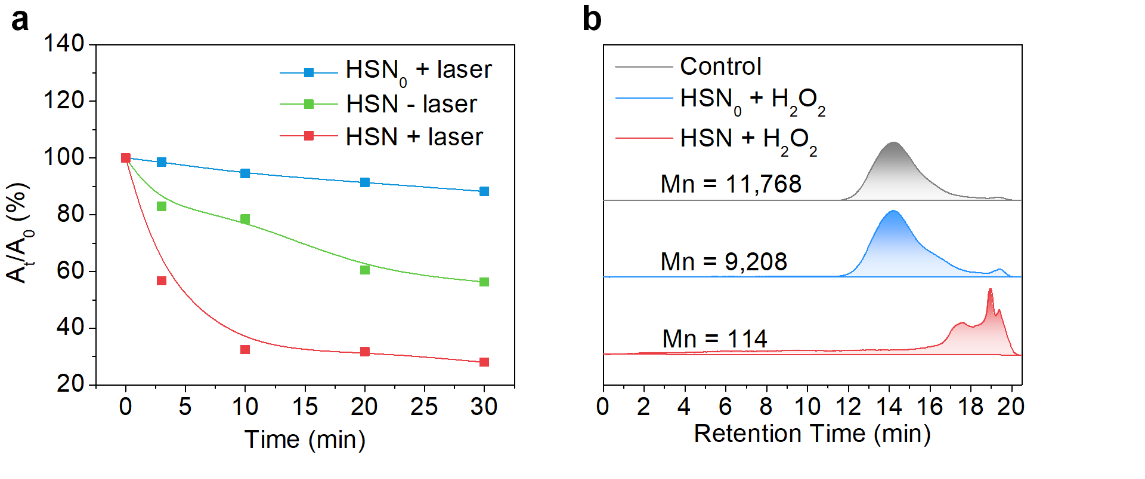


**Supplementary Figure 9.** Auto-degradation of HSN. (a) Time-course relative absorption (A_t_/A_0_% at 960 nm) of HSN or HSN_0_ in the presence of H_2_O_2_ (0.5 mM) with or without 1064 nm laser irradiation (1 W/cm^2^) (pH = 6.8). (b) Gel permeation chromatography result of HSN or HSN_0_ after auto-decomposition study under laser irradiation.


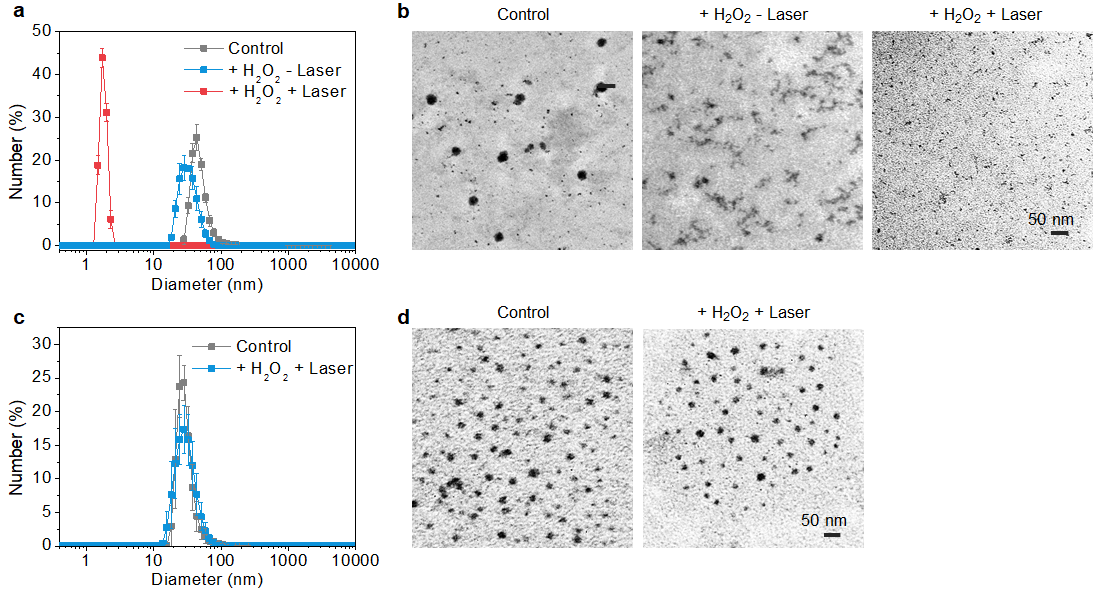


**Supplementary Figure 10.** DLS and TEM results of auto-degradation of HSN. (a) and (b): DLS and TEM results of HSN after H_2_O_2_ and laser treatments in Fig. S9a (laser irradiation: 10 min). (c) and (d) DLS and TEM results of HSN_0_ after H_2_O_2_ and laser treatments in Fig. S9a (laser irradiation: 10 min).


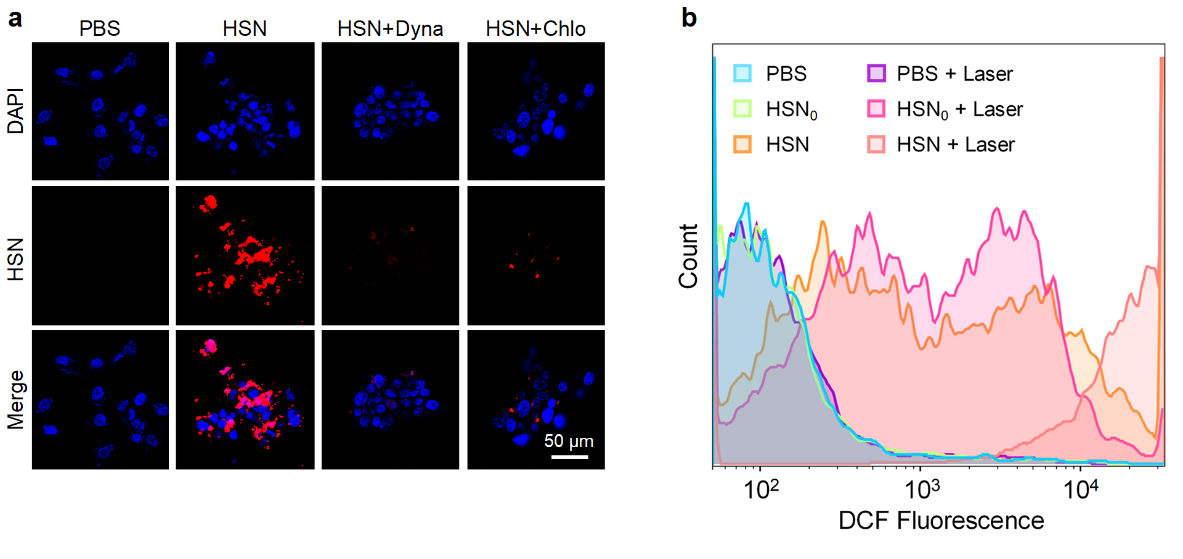


**Supplementary Figure 11.** (a) Confocal laser scanning microscopy images of 4T1 cells after respective incubation with PBS, fluorescently labelled HSN ([pTBCB] = 20 µg/mL), fluorescently labelled HSN together with endocytosis inhibitor Dynasore (Dyna, 50 µM) or Chloroquine (Chlo, 50 µM) for 24 h. Fluorescently labelled HSN was prepared by doping HSN with 5 w/w% nile red and detected with excitation at 561 nm and emission at 600-700 nm.. (b) Flow cytometry results of 4T1 cells after various treatments in Fig. 3D.


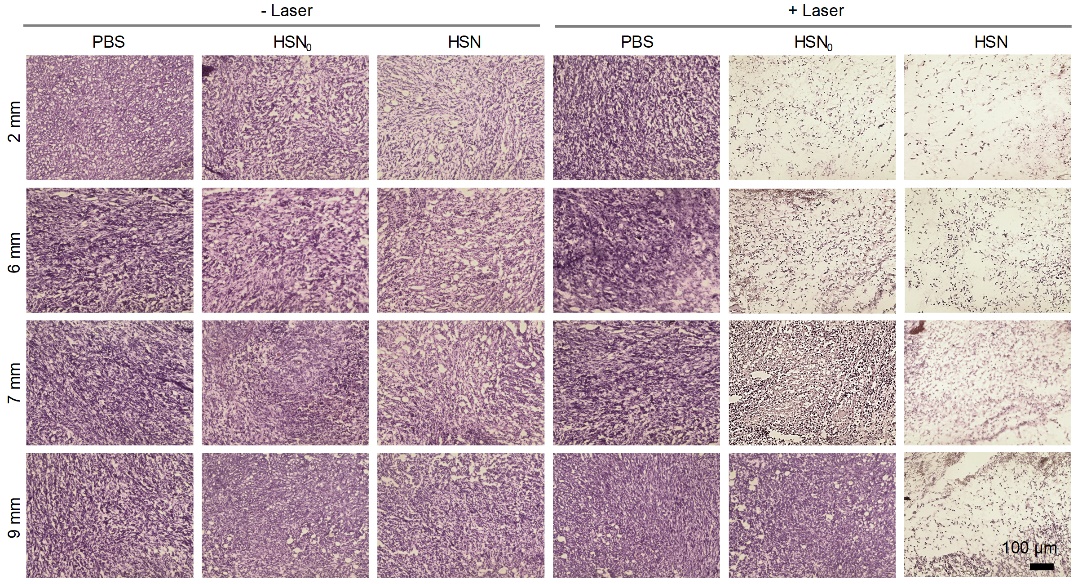


**Supplementary Figure 12.** H & E images of tumor sections. H &E staining was performed to tumor sections at different photothermal depths at 2 days after monotherapy or synergistic therapy.


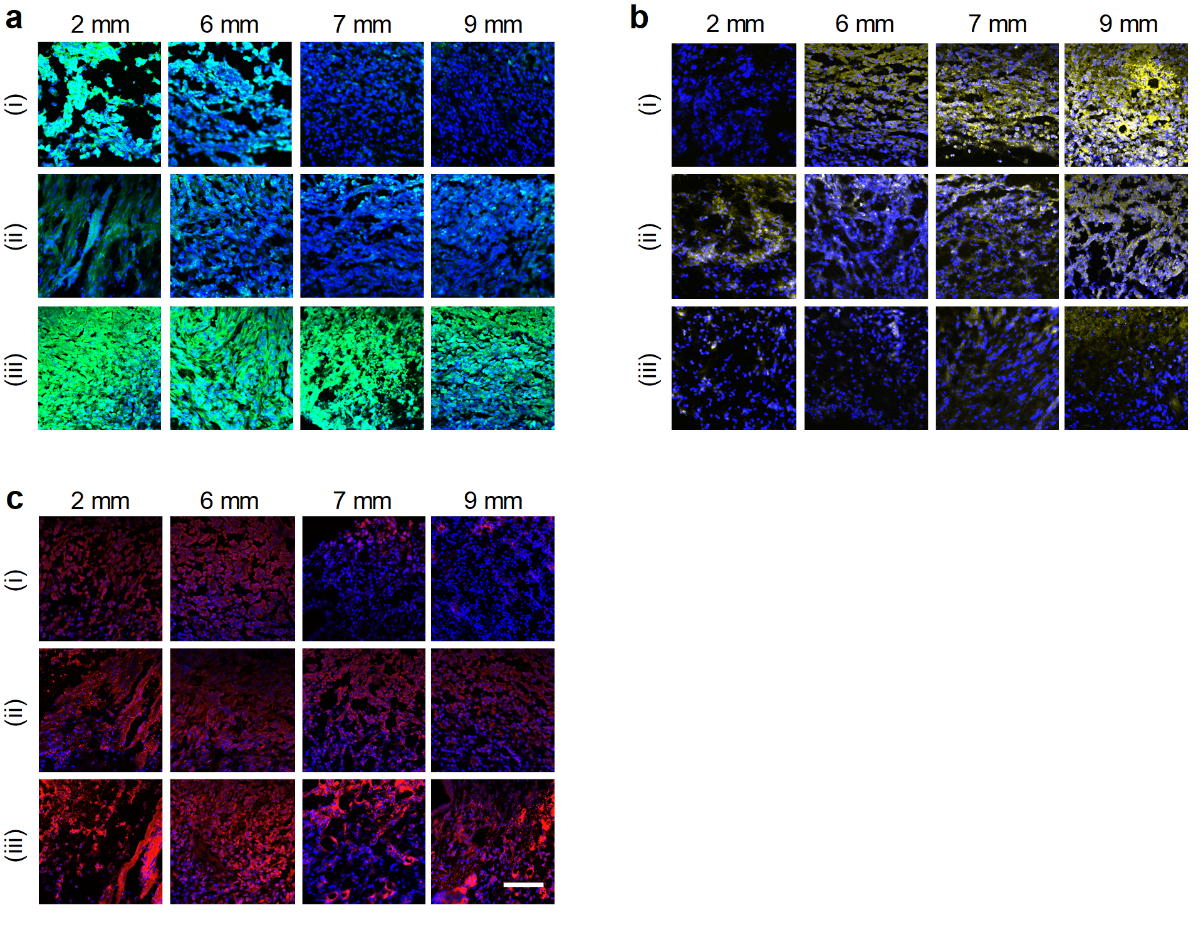


**Supplementary Figure 13.** Immunofluorescent staining of (a) Cas-3, (b) ACSL4, and (c) LPO staining of tumor sections. Staining was performed to tumor sections at different photothermal depths after monotherapy or synergistic therapy. Cas-3, LPO, and ACSL4 staining was indicated with green, red, and yellow false colors, respectively. (i) NIR-II PTT, (ii) ferrotherapy, (iii) NIR-II photothermal ferrotherapy.


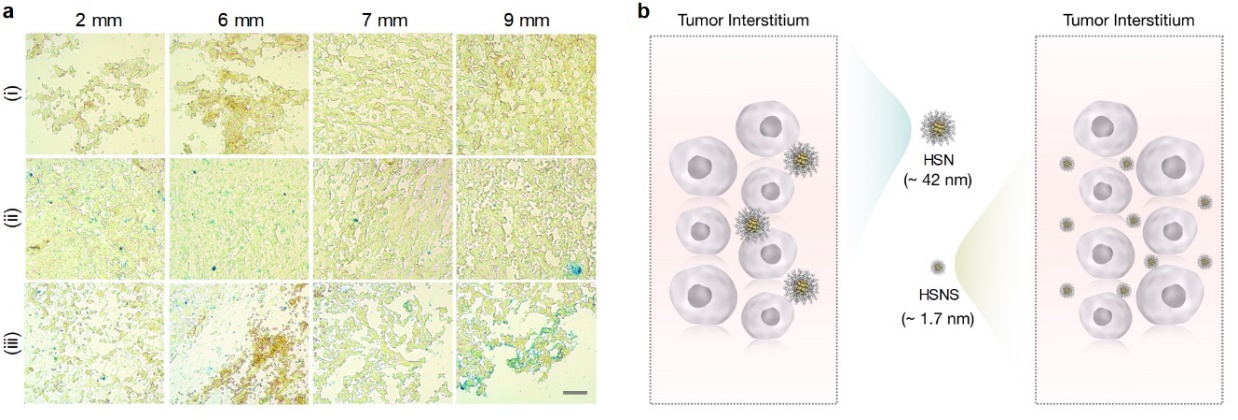


**Supplementary Figure 14.** Iron staining of tumor sections. (a) Iron staining of tumor sections at different photothermal depths after monotherapy or synergistic therapy. Scale bar: 50 µm. (b) Proposed scheme of permeability of HSN and HSNS in tumor interstitium. (i) NIR-II PTT, (ii) ferrotherapy, (iii) NIR-II photothermal ferrotherapy.


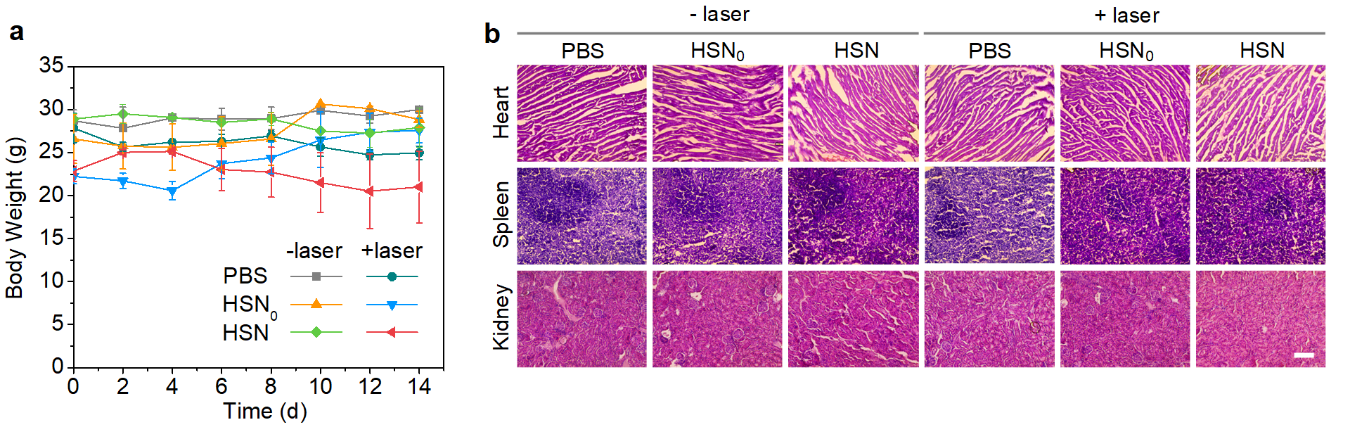


**Supplementary Figure 15.** Tumor growth curves and biocompatibility study. (a) Tumor growth curves of mice in Fig. 4D. (b) H & E images of major organs of mice in Fig. 4D at 14 days post-injection. Scale bar: 100 µm.


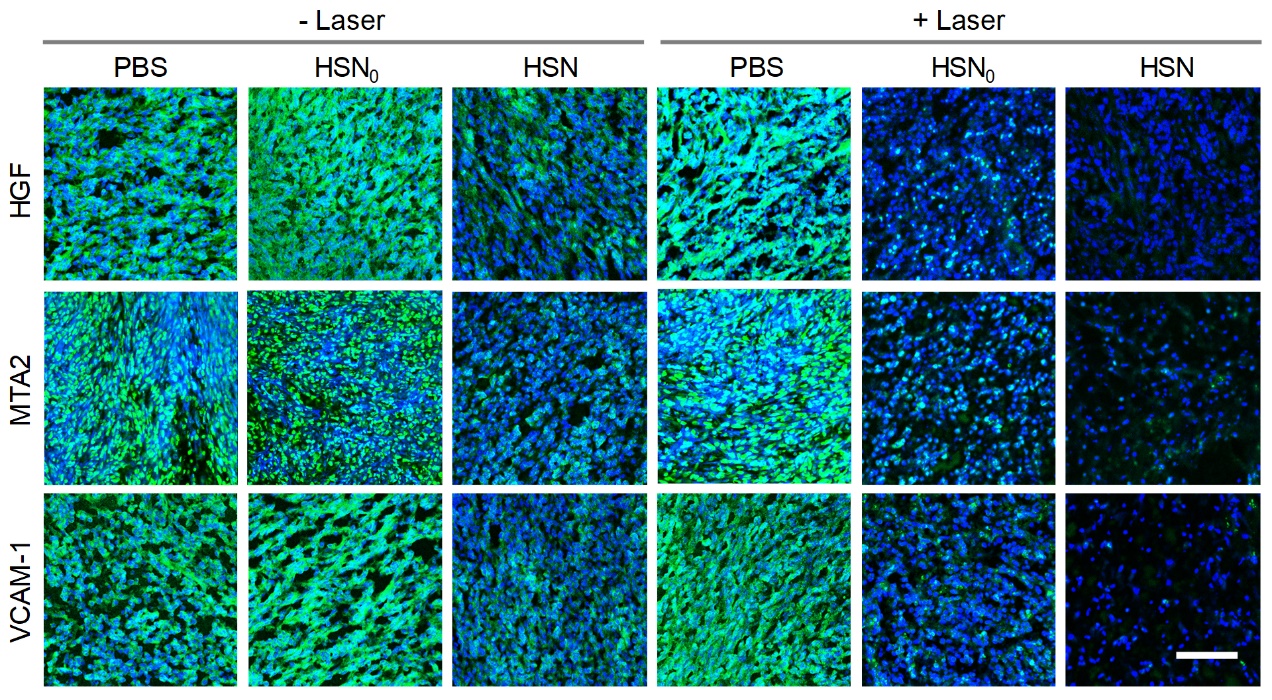


**Supplementary Figure 16.** Immunofluorescent staining of metastasis-related proteins in tumors. Green fluorescence indicated immunofluorescent staining of HGF, MTA2 and VCAM-1 of tumor tissues (at 9 mm) in living mice after 14 days of various treatments. Nuclei were stained with DAPI. Scale bar: 100 µm.
